# Atlas of regulated target genes of transcription factors (ART-TF) in human ES cells

**DOI:** 10.1186/s12859-022-04924-3

**Published:** 2022-09-16

**Authors:** Alexei A. Sharov, Yuhki Nakatake, Weidong Wang

**Affiliations:** 1 Laboratory of Genetics and Genomics, National Institute on Aging, National Institutes of Health, Baltimore, MD 21224-6825 USA; 2 Department of Systems Medicine, Mitsunada Sakaguchi Laboratory, Keio University School of Medicine, 35 Shinanomachi, Shinjuku-ku, Tokyo, 160-8582 Japan

**Keywords:** Genome binding of transcription factors, Induction of transcription factors, Regulated target genes, Parametric analysis of gene expression, Embryonic stem cells, Enhancer-promoter loop

## Abstract

**Background:**

Transcription factors (TFs) play central roles in maintaining “stemness” of embryonic stem (ES) cells and their differentiation into several hundreds of adult cell types. The regulatory competence of TFs is routinely assessed by detecting target genes to which they bind. However, these data do not indicate which target genes are activated, repressed, or not affected by the change of TF abundance. There is a lack of large-scale studies that compare the genome binding of TFs with the expression change of target genes after manipulation of each TF.

**Results:**

In this paper we associated human TFs with their target genes by two criteria: binding to genes, evaluated from published ChIP-seq data (*n* = 1868); and change of target gene expression shortly after induction of each TF in human ES cells. Lists of direction- and strength-specific regulated target genes are generated for 311 TFs (out of 351 TFs tested) with expected proportion of false positives less than or equal to 0.30, including 63 new TFs not present in four existing databases of target genes. Our lists of direction-specific targets for 152 TFs (80.0%) are larger that in the TRRUST database. In average, 30.9% of genes that respond greater than or equal to twofold to the induction of TFs are regulated targets. Regulated target genes indicate that the majority of TFs are either strong activators or strong repressors, whereas sets of genes that responded greater than or equal to twofold to the induction of TFs did not show strong asymmetry in the direction of expression change. The majority of human TFs (82.1%) regulated their target genes primarily via binding to enhancers. Repression of target genes is more often mediated by promoter-binding than activation of target genes. Enhancer-promoter loops are more abundant among strong activator and repressor TFs.

**Conclusions:**

We developed an atlas of regulated targets of TFs (ART-TF) in human ES cells by combining data on TF binding with data on gene expression change after manipulation of individual TFs. Sets of regulated gene targets were identified with a controlled rate of false positives. This approach contributes to the understanding of biological functions of TFs and organization of gene regulatory networks. This atlas should be a valuable resource for ES cell-based regenerative medicine studies.

**Supplementary Information:**

The online version contains supplementary material available at 10.1186/s12859-022-04924-3.

## Background

Regulation of the rates of transcription of various genes is the key component of gene regulatory networks in living cells. Most regulatory pathways such as signal transduction and metabolic homeostasis are mediated by the activation of transcription factors (TFs) that bind to target genes and change the rate of their transcription [1–3]. TFs bind DNA in a sequence-specific way, and binding sites of TFs were initially mapped based on short DNA motifs identified with HT-Selex [4, 5] and other methods. Later in the last two decades, the binding capacities of many TFs have been extensively explored thanks to a new technology of massively parallel sequencing of short DNA fragments extracted via immunoprecipitation of crosslinked chromatin (ChIP-seq) [1, 6] and DNase-seq [7, 8]. The study of TF binding sites on DNA has extended considerably our knowledge of TFs.

In contrast, the progress in the study of the regulatory role of TFs after their binding to DNA is lagging behind and has not been supported by high-throughput methods. It has been reported that the majority of binding sites of TFs are not associated with the change of expression of nearby genes [9–11]; thus, the information on the genome location of binding sites appears not sufficient for predicting the regulatory role of corresponding TFs (e.g., direction and strength of gene expression change). Some regulatory effects can be identified from the comparison of gene expression profiles in the wild type and knock-out (KO) cells [12, 13], however, this approach is not always reliable. Knock-out cell lines may carry additional changes in their genomes besides the disrupted TF, and the effects of the disrupted TFs are often compensated by alternative signalling pathways. Also, these compensatory mechanisms may result in a dramatic change of expression of many genes that are not targets of the disrupted TF. To overcome these problems, it is necessary to use transient manipulations of TFs followed by global gene expression profiling of cells shortly after the TF was either induced or repressed [14]. This method is labour-intensive, and thus usually applied to a single TF or a small group of related TFs. Large scale projects of transient manipulation of individual TFs are rare [14–19].

In this paper we present an atlas of regulated targets of TFs (ART-TF) in human ES cells by combining data on TF binding with a large-scale study of the gene expression change after induction of individual TFs in human ES cells [19]. Results of experiments on binding and regulatory capacities of TFs are integrated to find downstream target genes that are bound and then either activated or repressed by a TF in a specific cell type. Because the notion of “target gene” often refers solely to the binding capacity of TFs, we introduce here a new term “regulated target gene” which denotes a gene that is not only bound by a TF but also regulated by the TF in a specific way.

Taking a simple overlap of sets of genes that are bound and regulated by a TF is not a reliable approach for identifying sets of regulated target genes because sets of genes may intersect by pure chance. In this paper we use a statistical method for delimiting regulated target genes as a subset within the overlap of these sets, which guarantees that the proportion of false positive genes (i.e., intersecting by chance) is less than a specified threshold [11, 20]. This method, called the Expected Proportion of False Positives (EPFP), was further elaborated here to accommodate additional information on the scores of individual target genes (see Methods).

## Results

### Enrichment of TF targets among genes that responded to TF induction

To explore the association between two main functions of TFs, which are sequence-specific binding to genomic DNA and regulating the transcription rate (expression) of genes located in the vicinity of binding sites, we analyzed the association of individual TFs with its target genes by two criteria: binding to the genome near target genes and changing the expression of target genes shortly after forced induction of each TF. The first criterion was assessed by using publicly available ChIP-seq data (*n* = 1868) for 311 TFs (Additional file 1), and the second criterion was evaluated from a recent large-scale experiment on the induction of 510 individual TFs in human ES cells with subsequent global gene expression profiling using a combination of RNA-seq and microarray experiments 48 h after TF induction [19]. Multiple ES cell clones carrying doxycycline (Dox)-inducible transgenes of each TF were generated and then used for upregulation of these TFs by adding Dox to the medium. By induction of a TF, we mean the increase of mRNA gene expression followed by increased protein synthesis of a specific TF. Protein synthesis is confirmed by visualizing the expression of an IRES-LacZ reporter connected immediately after the transgene in the vector transfected to all ES cell clones, which was normally observed in almost 100% of cells, as well as by immunostaining in a subset of clones [19]. The increased abundance of TF proteins does not necessarily result in increased activity, which can be affected by protein modification or interactions with cofactors; however the increased activity of a TF can be inferred from the subsequent upregulation of its target genes.

**Fig. 1:**
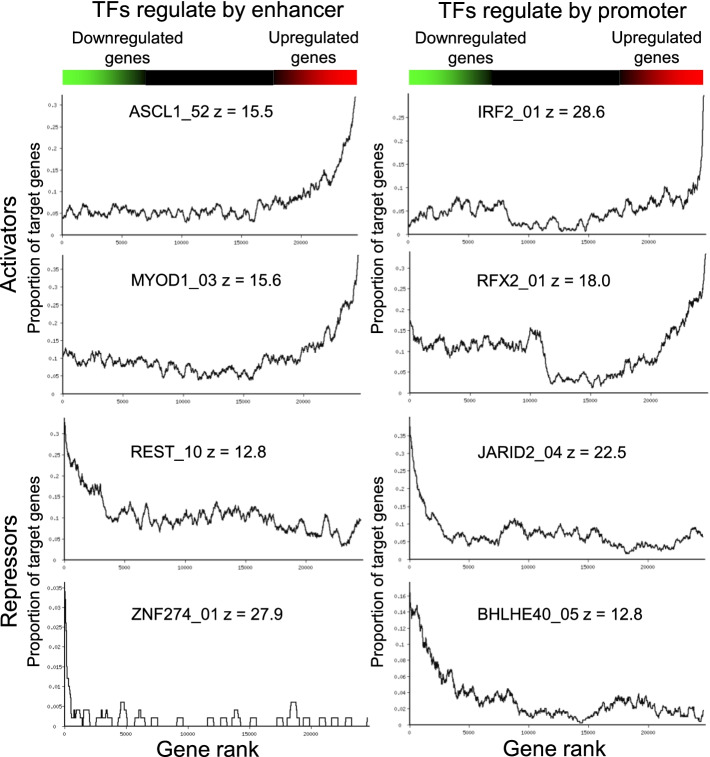
Rank-plots showing the enrichment of targets among either upregulated or downregulated genes after TF induction. The proportion of target genes is estimated in a sliding window of 300 genes sorted by their expression change after TF (color bar at the top); each plot is annotated by ChIP-seq experiment name and z-value for the PAGE gene set enrichment test (Additional file 1)

Rank order plots (rank-plots) [20] were used to visualize the enrichment of targets (genes bound by a TF) among genes that changed expression following the induction of the TF (Fig. 1). Genes were sorted by their expression change after induction of the TF (downregulated genes are on the left and upregulated on the right), and the proportion of target genes (i.e., bound by the same TF) was estimated in a sliding window of 300 genes. Genes upregulated after the induction of ASCL1, MYOD1, IRF2, and RFX2, show an increased proportion of TF targets at the right side, indicating that they were activated by TF binding. Genes downregulated after the induction of REST, ZNF274, JARID2, and BHLH40, show an increased proportion of TF targets at the left side, indicating that they were repressed by TF binding. This is consistent with the repressing function of these TFs [21–24].

The enrichment of targets among genes that responded to TF induction was statistically evaluated using PAGE method [25], which was modified by applying it separately to upregulated and downregulated genes, and accounting for scores of individual binding sites in ChIP-seq data; it was estimated with ExAtlas [20]. All *z*-values for rank-plots in Fig. 1 are far greater than 2, and thus are statistically significant. The distribution of *z*-values generated by PAGE (maximum of four combinations of promoter/enhancer and upregulation/downregulation in the Additional file 2) shows significant gene set enrichment (*z* ≥ 2) for 1455 out of 1833 ChIP-seq experiments for TFs matching the induced TF in ES cells. The average *z*-value among significant ChIP-seq data is 5.91. Analysis of 1454 ChIP-seq experiments yielded one or more regulated target genes (i.e., 79.3% success rate).

### Comparison of methods for delineating regulated target genes of TFs

We compared the effectiveness of three methods used for delineating “direct” regulated target genes, where the induced TF was the same as the one used in ChIP-seq experiment. Method #1 employed separate analysis of TF proximal binding sites in promoters (from −500 to +500 bp from TSS) and distal binding sites in enhancers (from −100 to +100 Kb from TSS, excluding promoter), and estimated the score of each target gene as a sum of scores of all associated binding sites. Here, by enhancer we simply mean a distal binding site of a TF rather than published gene regulatory regions identified with experimental and computational approaches [26–28]. Method #2 also used separate analysis of proximal and distal binding sites, but the score of a target gene was equal to the maximum score among associated binding sites. Method #3 did not distinguish proximal and distal binding sites, and used the sum of scores of all associated binding sites. For all three methods we used gene enrichment analysis (PAGE) with ExAtlas [20], EPFP threshold of 0.30, and fold change threshold of 1.5.

**Fig. 2:**
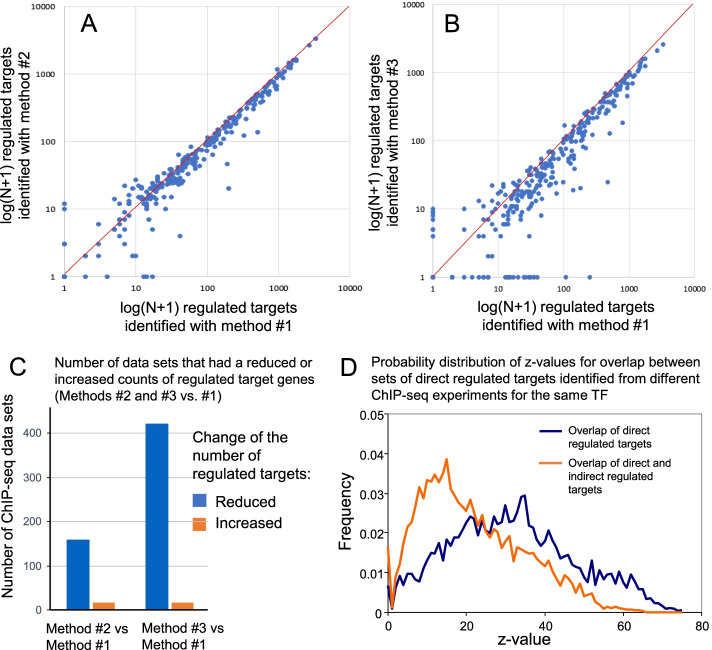
Comparison of methods for delineating regulated target genes of transcription factors (TFs). **A**, **B** Counts of direct regulated target genes (total upregulated, downregulated, promoter- and enhancer-dependent) identified by 3 methods described in “Comparison of methods for delineating regulated target genes of TFs” section; each point is a single TF. **C** Number of TFs that had a reduced or increased counts of direct regulated target genes identified with methods #2 and #3 versus #1. **D** Probability distribution of z-values (hypergeometric test) that represent the significance of overlap between direct regulated targets identified from different ChIP-seq experiments for the same TF (blue) and between direct and indirect regulated targets for the same set of TF (orange)

The number of identified regulated target genes tended to be greater for method #1 than for methods #2 and #3 for the majority of TFs (Fig. 2A, B). Method #1 yielded a significantly greater number of regulated targets (*p* ≤ 0.001, chi-square test) for 158 and 421 ChIP-seq data sets as compared to methods #2 and #3, respectively. In contrast, only 15 and 16 ChIP-seq data sets have significantly smaller number of regulated targets generated by method #1 as compared to methods #2 and #3, respectively (Fig. 2C). Because method #1 was more successful for delineating regulated target genes for most TFs, we used it for further analysis.

Also, we tested if regulated target genes can be predicted from inferred binding sites identified from ChIP-seq data with an antibody to a different (i.e., surrogate) TF, which either belongs to the same gene family as the induced TF, or interacts with the induced TF (Additional file 3). In both cases, it was expected that many binding sites of a surrogate TF are co-localized with binding sites of the induced TF, and thus can be used as an indirect evidence of binding. We call regulated target genes “indirect” if they were identified from surrogate ChIP-seq data. The significance of overlap between sets of direct and indirect regulated targets for the same TF was quantified by the hypergeometric test (z-value). The overlap between sets of direct and indirect regulated targets was generally lower than the overlap between sets of direct regulated targets identified using different ChIP-seq data for the same TF, as follows from the probability distribution (Fig. 2D). This means that direct ChIP-seq data have a higher quality for finding targets of TFs than surrogate ChIP-seq data, as expected. But the median *z*-value for the overlap of indirect and direct regulated targets is still highly significant (*z* = 21.6, *p* < 10^–70^), and thus, indirect regulated targets can still be used for examining the regulatory network links and functions of TFs. The total number of regulated targets of TFs was increased by 63% after we added indirect regulated targets to the database.

Another potential problem is the type of cells used in ChIP-seq experiments. From the theoretical point of view, the best approach would be using the same cell type for both TF induction and ChIP-seq experiments, which in our case is pluripotent ES cell. However, several practical problems indicate that limiting the analysis to only those ChIP-seq experiments that used ES cells is not always the best option for all TFs. The first issue is that only 6.08% (N = 115) of compiled ChIP-seq data were done with ES cells, and these data represent just 57 TFs, of which only 37 TFs have multiple replications in ES cells that yielded sets of regulated target genes. The second issue is that many TFs related to cell differentiation are not expressed in ES cells and therefore cannot be captured by the standard ChIP-seq method. Finally, the third issue is that the timing of ChIP-seq experiments is very different from the induction of TFs in cultured cells. The ChIP-seq assay captures the instantaneous state of cells, whereas the induction of TFs is a long process (48 h, in our case), where the state of cells is continuously perturbed. Therefore, after a few hours of TF induction, the binding locations of a TF may change as cells get differentiated and are no longer in a pluripotent state. Thus we suggest that published ChIP-seq data for differentiated or partially differentiated cells may yield more relevant information on TF binding sites in cells derived from ES cells via induction of TFs than published ChIP-seq data obtained with ES cells.

**Fig. 3:**
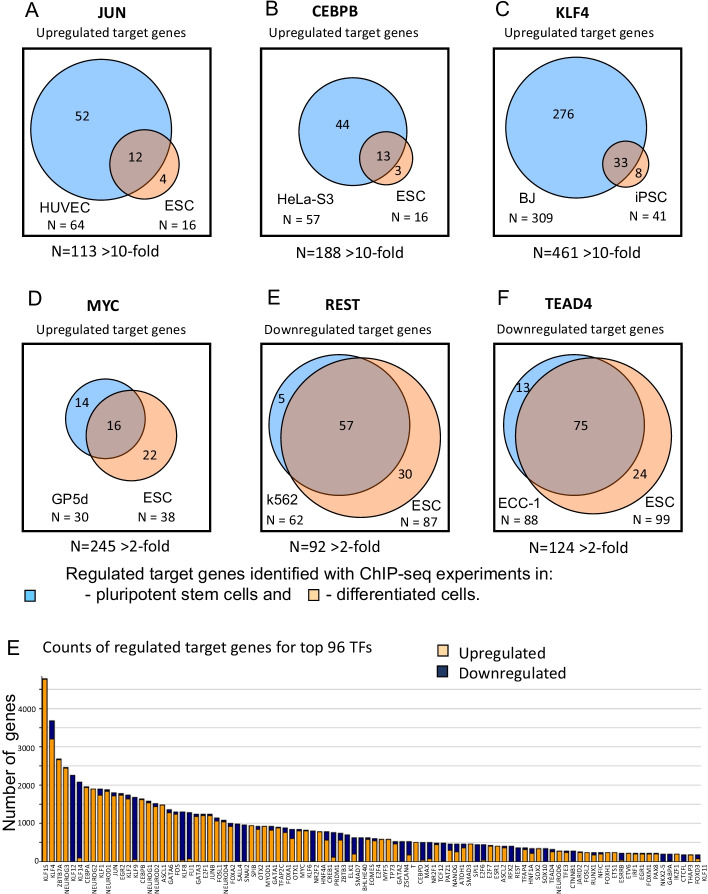
Sets of regulated target genes for human TFs. **A**–**F** Examples of sets of regulated target genes identified with ChIP-seq data coming from pluripotent stem cells (ES or iPS cells) or from differentiated cells. Squares are sets of regulated target genes with largest gene expression change (≥ tenfold or ≥ twofold) after induction of six TFs: JUN, CENPB, KLF4, MYC, REST, and TEAD4, combined from all available ChIP-seq data. Subsets of regulated target genes identified from ChIP-seq data in pluripotent stem cells and in differentiated cells are shown by circles colored orange and blue, respectively. **E** Counts of regulated target genes for top 96 TFs

Here we present several typical examples of results obtained with ChIP-seq data from pluripotent stem cells (e.g., ES cells) versus those from differentiated cells. In Fig. 3A–F, we used the size of squares to represent the number of regulated target genes that strongly changed their expression (≥ tenfold in top row and ≥ twofold in bottom row) after induction of six representative TFs. These regulated target genes were compiled from all available ChIP-seq data for each of these TFs as explained in “Compiling sets of regulated target genes of TFs and comparison with existing databases” section. The size of circles represents the number of regulated target genes identified from one specific ChIP-seq experiment with either pluripotent (orange) or differentiated cells (blue). ChIP-seq data on binding of JUN, CENPB, and KLF4 in pluripotent stem cells points to only a small portion of target genes that are upregulated following induction of these TFs (orange circles), whereas ChIP-seq data in differentiated cells points to a much larger portion of upregulated target genes (blue circles). This means that data on binding of TFs in differentiated cells appears much more informative in predicting regulated target genes than binding of these TFs in pluripotent stem cells. In contrast, the gene regulation effect of repressing TFs, REST and TEAD4, is better predicted by ChIP-seq data in pluripotent stem cells than in differentiated cells (Fig. 3E, F). Gene regulation by MYC shows an intermediate pattern, where blue and orange circles complement each other. These examples show that to understand regulation of gene expression, the best approach seem to be integrating ChIP-seq data from many different cell types.

### Compiling sets of regulated target genes of TFs and comparison with existing databases

The counts of regulated target genes were combined for all ChIP-seq experiments, including proximal and distal binding sites of the same TF. Because the most reliable regulated target genes are those that are supported by multiple ChIP-seq data sets, the regulated target gene candidates supported by a single ChIP-seq experiment were not included in our final list of genes, except for 75 TFs for which only one ChIP-seq data set yielded some regulated targets.^1^ Lists of direction- and strength-specific regulated target genes are generated for 311 TFs (out of 351 TFs tested) with expected proportion of false positives EPFP ≤ 0.30 (Additional file 4 and Additional file 5). We expect that our data will be used by researchers with different objectives; some of them are interested only in direct regulated targets, whereas others may prefer combined data from direct and indirect ChIP-seq experiments. Thus, we specify for each regulated target gene if it is derived only from indirect ChIP-seq experiments (Additional file 5).

Counts of regulated target genes for top 96 TFs are shown in Fig. 3E. The highest number of regulated targets was found for KLF15 (n = 4796). Strong activation effects are seen in KLF15, KLF4, ZBTB7A, NEUROG3, CEBPA, and NEUROG2 whereas strong repression effects appear in KLF12, KLF14, KLF9, FLI1, KLF8, and SALL4.

In contrast to our approach, most existing databases of targets of TFs (ENCODE, JASPAR, and TFTG_DB) [29–31] are based solely on binding sites identified via ChIP-seq, binding motifs (e.g. HT Selex), or DNase footprints, and do not consider the direction and strength of regulation effects. Only TRRUST database [32] considers the direction of gene expression change, and thus is a competitor of our ART-TF. We used the hypergeometric test to evaluate if sets of target genes of the same TF regulated in the same direction taken from TRRUST and ART-TF overlap stronger than expected by random. It appears that only 18 sets of upregulated target genes (out of 148 sets of upregulated genes and 131 sets of downregulated genes) matched significantly (*p* ≤ 0.05) between TRRUST and ART-TF for the same TF and direction of gene expression change (Additional file 6). In TRRUST, the sets of regulated target genes (upregulated + downregulated) are mostly smaller than in ART-TF: out of 190 common TFs, TRRUST has 37 TFs (19.5%) with larger sets of regulated target genes, whereas ART-TF has 152 TFs (80.0%) with larger sets of regulated target genes. Also, ART-TF has 124 new TFs that are not present in the direction-specific part of TRRUST, among which 63 TFs are also not found in ENCODE, JASPAR, TRRUST and TFTG_DB (Additional file 6).

### Asymmetry in activating and repressing effects of TFs

**Fig. 4:**
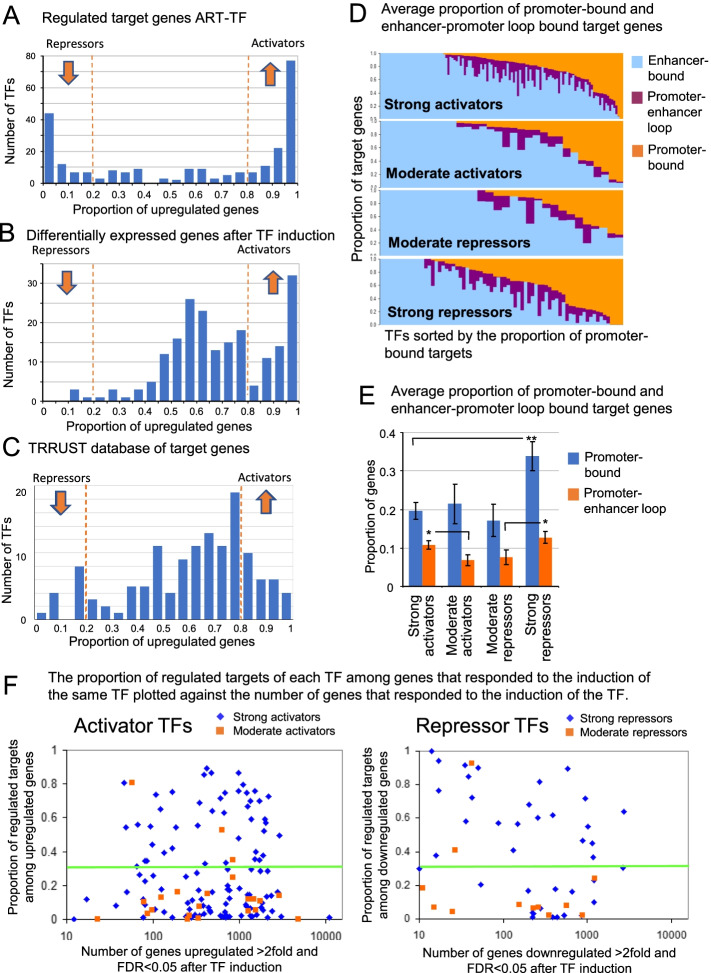
Activating and repressing effects of TFs. **A** Number of TFs with varying proportion of upregulated target genes after TF induction. **B** Number of TFs with varying proportion of upregulated genes after TF induction (> twofold, FDR < 0.05). **C** Number of TFs with varying proportion of upregulated genes in the TRRUST database. **D** The proportion of target genes regulated by binding of individual TFs to enhancers, promoters, and enhancers + promoters in four groups of TFs: strong activators, moderate activators, moderate repressors and strong repressors. **E** Average proportion of promoter-bound and enhancer-promoter loop bound target genes in the same four groups of TFs as in (**D**). Vertical lines show SD based on one-way ANOVA; statistical significance of pairwise comparison of means: (*) p < 0.05, (**) p < 0.01. **F** The proportion of regulated targets of each TF among genes that responded to the induction of the same TF (> twofold change, FDR < 0.05) plotted against the number of genes that responded to the induction of the TF. Upregulated genes are shown for activator TFs and downregulated genes—for repressing TFs

Many TFs specialize in either activating or repressing functions [1]. Thus, it was interesting to compare the proportion of upregulated genes among target genes regulated by TF binding, and among all genes whose expression changed after the induction of TFs. We called TFs strong activators (or repressors) if the proportion of upregulated target genes after TF induction, *q*, was ≥ 80% (or ≤ 20%) (Additional file 4). Other TFs were classified as either moderate activators (if 50% ≤ q < 80%) or moderate repressors (if 20% < q < 50%). Sets of regulated targets show strong asymmetry in their response to TF induction: the majority of TFs are either strong activators (N = 119, 47.0% out of 253 TFs with ≥ 10 regulated targets) or strong repressors (N = 71, 28.1%), and only 62 TFs (24.5%) are moderate activators or repressors in the middle (Fig. 4A). In comparison, the distribution of the proportion of upregulated genes among all genes that were affected by the induction of TFs (twofold change, FDR ≤ 0.05) has a weaker asymmetry (Fig. 4B). The majority of induced TFs (N = 135, 67.2%) had no clear prevalence between activation and repression effects with a proportion of upregulated genes between 20 and 80%. Strong activation effect (≥ 80%) is observed in 62 TFs (30.8%), and strong repressing effect—only in 4 TFs (2.1%). Thus, the abundance of regulated target genes is a better indicator of activating and repressing effects of TFs than the number of upregulated and downregulated genes following manipulation of TFs. TRRUST database does not show asymmetry in activating and repressing effects of TFs (Fig. 4C): the frequency distribution of the proportion of activated genes is bell-shaped with only few TFs that are strong activators or strong repressors. The lack of asymmetry in TRRUST possibly resulted from assembling data from studies on various cell lines and tissues, whereas data in ART-TF comes from one cell type (ES cells).

### Transcription regulation by binding of TFs to enhancers and promoters

Distinguishing of TF binding to promoters and enhancers of genes is not trivial because activated enhancers are connected to promoters by mediator, cohesin, and other proteins making a DNA loop [33, 34]. ChIP-seq procedure used for detecting TF binding sites includes a crosslinking step that enables a covalent connection between interacting proteins and DNA, and thus, may include DNA fragments from both enhancer and promoter. In our analysis of TF-regulated targets we distinguish 3 situations, where (1) binding site was only in the promoter, (2) only in enhancer, and (3) both in the promoter and enhancer. We estimated the proportion of each situation for target genes regulated in the dominant direction (i.e. upreglated for activator TFs and downreguated for repressors) (Fig. 4D).

Most human TFs (N = 207, 82.1%out of 252 TFs with ≥ 10 regulated targets) bind to enhancers (sometimes combined with binding to promoters) of at least half of their regulated target genes (Additional file 4). A smaller set of TFs (N = 45, 17.9%) bind the majority of target genes exclusively in promoters. Examples of TFs that activate target genes via promoter binding are cell cycle-related genes (E2F1, E2F4, E2F5, FOXM1, MYC, MYCN), immune-related genes (SPIB, IRF1, IRF5, STAT3), and insulators (CTCF, CTCFL). Examples of TFs that repress target genes via binding to promoters are SNAIL proteins (SNAI1, SNAI2, SNAI3), cell cycle repressors (E2F6, E2F7, MAX), and others (e.g., FLI1, ELK1, UBTF, GABPA, HEY, and HES). The average proportion of target genes regulated by binding of TFs to promoters alone is significantly higher (33.8%, *p* < 0.01, ANOVA) among strong repressors, than in strong activators (19.6%) (Fig. 4E). Thus, repression effects of TFs are more often mediated by promoter-binding than activation effects.

Binding of TFs to both enhancer and promoter was detected in 10.4% of target genes regulated in the dominant direction, in average (Additional file 4). Strong combined enhancer-promoter binding (> 30% of regulated targets) was identified in some repressors (e.g., KLF12, KLF14, KLF9, TEAD4, JARID2, ZNF274) and activator TFs (e.g., KLF15, KLF4, ZBTB7A, NEUROG3, NEUROG2). These TFs likely participate in the formation of enhancer-promoter DNA loops. The average proportion of target genes with combined enhancer-promoter binding was higher among strong activator TFs and strong repressor TFs, as compared to moderate activators and moderate repressors, respectively (Fig. 4E) (*p* < 0.05, ANOVA).

### Explanatory power of information on regulated target genes

The explanatory power of studying target genes of TFs can be demonstrated by showing that target genes of each TF comprise a large proportion among all genes whose expression change significantly after induction of this TF. By significant gene expression change we mean criteria developed by Nakatake et al. [19]: ≥ twofold change in relation to 3 controls: same cell line without Dox and two cell lines with neutral transgenes (Emerald and rtTA3G) cultured with Dox, and false discovery rate FDR < 0.05. For simplicity we focus on the dominant direction of gene expression change: upregulation of target genes – for activator TFs, and downregulation – for repressor TFs.

The proportion of regulated targets among responding genes reached such high values as 89% for activators and 100% for repressors, and does not show a dilution effect with increasing number of responding genes (Fig. 4F). In average, 30.9% of genes that respond ≥ twofold to the induction of TFs are regulated targets (Additional file 4). Sets of regulated target genes for 50 activator TFs and 27 repressor TFs are sufficiently informative because they comprise ≥ 30% of genes significantly affected by TF induction (Fig. 4F, above the green line). Most of these TFs were either strong activators (e.g., FOS, JUN, NEUROD1, NEUROG3, ASCL1, GATA3, MYC, KLF15, E2F1) or strong repressors (e.g., REST, SMAD7, SNAI2, SALL4, KLF14, E2F7). The proportion of TFs with sufficiently informative sets of target genes is 41.0% among TFs with ≥ 10 genes affected by their induction (n = 77 out of 188 TFs that cause expression change in ≥ 10 genes). It is highest among strong repressors (64.1%, n = 25 out of 39) and strong activators (40.9%, n = 47 out of 115), and substantially lower among moderate repressors (16.7%, n = 2 out of 12) and moderate activators (13.6%, n = 3 out of 22). The proportion of regulated targets in a set of genes affected by TF induction averaged over all TFs was 30.9%. In particular, there are 54 TFs with regulated targets comprising ≥ 50% of genes affected by TF induction; which we consider an indicator of success of our method.

### Similarity of sets of regulated targets between TFs

**Fig. 5:**
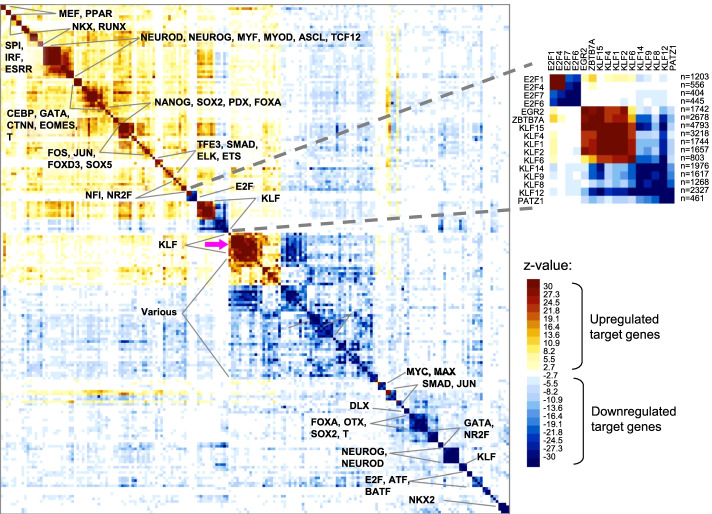
Similarity of sets of regulated targets of 150 TFs evaluated by the enrichment of common genes (z-values from the hypergeometric test). For downregulated genes, z-values are multiplied by (− 1). TFs with weak similarity of their regulated targets are not shown. Details for E2F and KLF factors are shown in the inlet, where “n” is the number of regulated target genes. For details see Additional file 7 and Additional file 8

To provide a bird view on the sets of regulated targets in ART-TF we generated a similarity matrix indicating the enrichment of common (i.e., overlapping) genes in comparison with expected overlap in random sets using hypergeometric test in ExAtlas [20]. Upregulated and downregulated target genes for each TF were analyzed as separate sets. Z-values were multiplied by (-1) for downregulated sets of genes, to distinguish them visually from upregulated sets of genes. The matrix of z-values (Fig. 5) (Additional file 7 and Additional file 8) shows high similarity between sets of upregulated targets for TFs that belong to the same gene family, such as LHX, MEF, NKX, RUNX, and ESRR. Groups of TFs with similar upregulated target genes also corresponded to comparable roles in cell differentiation. For example, upregulated target genes of NEUROD, NEUROG, MYF, MYOD, ASCL, and TCF12 are similar because they support cell differentiation to neural and muscle lineages, whereas similarity of upregulated target genes of CEBP and GATA follows from the role of these TFs in differentiation of cells towards hematopoietic lineages.

Interestingly, some TFs from the same gene family cause opposite effects in regulation of their common target genes. For example, E2F1 and E2F4 are activators whereas E2F6 and E2F7 are repressors of overlapping sets of genes (Fig. 5, the right inlet). Both repressors E2F6 and E2F7 lack transcriptional activation domain in contrast to E2F1 and E2F4 that carry activation domain [35–37]. Also, repressive role of E2F7 is consistent with its capacity to recruit CtBP that inactivates E2F1 via dimer formation [36]. The repressing effect of E2F6 is achieved by binding to polycomb-group proteins or via the formation of a complex that includes MGA and MAX proteins [38, 39]. Based on our data, E2F4 is an activator in human ES cells, although it has been reported previously as repressor in other cell types [37].

A similar combination of activating and repressing effects was observed in members of the KLF gene family: KLF1, KLF2, KLF4, KLF6, and KLF15 are strong activators, and KLF8, KLF9, KLF12, and KLF14 are strong repressors of a similar set of target genes when induced in ES cells (Fig. 5, the right inlet). This difference is explained by the fact that activating KLF factors carry no CtBP or Sin3a binding sites that mediate interaction with repressors, whereas KLF8, and KLF12 have CtBP sites, and KLF9 and KLF14 have Sin3a sites [40]. Repressor TFs KLF8, KLF9, KLF12, and KLF14 also have a weak activation effect upon an entirely different set of genes (a block of activation effects pointed by magenta arrow in Fig. 5). The mechanism of this effect is unknown. Thus, opposite activation/repression effects within members of the same TF family (E2F and KLF) can be explained by their structure and interaction with partner proteins.

## Discussion

Our study contributes to solving the problem of combining information on TF binding to promoters and enhancers of target genes with independent data on the response of TF target genes to the manipulation of individual TFs. We developed new statistical methods and applied them to compare published data on DNA binding of TFs (1981 ChIP-seq data) with a large-scale database of the gene expression change immediately after induction of individual TFs in human ES cells [19].

The main result of the paper is that we have compiled a new and more complete atlas of regulated targets of TFs (ATR-TF) in human ES cells. This database provides additional direction-specific regulated targets that complement the existing TRRUST database, and partially overlaps with it. We identified regulated target genes for 311 TFs, including 123 new TFs not present in the direction-specific portion of TRRUST (63 of them are new for ENCODE, JASPAR, TFTG_DB, and TRRUST). Also, sets of regulated targets for 152 TFs were expanded in comparison to TRRUST (i.e., 80.0% of 190 common TFs in ART-TF and TRRUST). For some TFs, we used surrogate ChIP-seq data from TFs that differ from the manipulated TF on the basis that they either belong to the same gene family or interact with the manipulated TF and share the binding site. The use of surrogate data allowed us to add 63% of regulated target genes. The atlas of regulated target genes is a valuable bioinformatics resource because it allows biologists to explain the mechanism of expression change in 30.9% genes (in average) that responded to the induction of TFs in human ES cells.

Analysis of sets of regulated targets showed that most studied TFs are either strong activators or strong repressors. But this asymmetry in activation/repression effects is less pronounced in the counts of upregulated and downregulated genes after TF induction. Some families of TFs (e.g., E2F and KLF) include both activators and repressors and these effects depend on the presence of activation domains or binding sites of repressors in their protein structure.

Most human TFs (82.1%) regulate their target genes via binding to enhancers (which can be combined with promoter binding). Repression effects are more often mediated by exclusive promoter-binding than activation effects. Regulation via promoter is apparently faster, and thus, it is involved in such functions as cell-cycle and immune response that require immediate activation or repression [41]. Binding of TFs to both enhancer and promoter was detected in 10.4% of regulated target genes, and possibly indicates the involvement of TFs in enhancer-promoter DNA loops. Our data indicates that enhancer-promoter loops are more abundant among strong activator TFs and strong repressors than in moderate activators and repressors. We believe that functional analysis of TFs provides new insights into the roles of many TFs in cellular metabolism that can be tested experimentally in the future. In particular, this information may be helpful in regenerative medicine for guided differentiation of pluripotent cells into specialized cell types [42, 43].

Naturally, our study has some limitations which are necessary to mention here. First, manipulation of TFs was done in only one cell type: ES cells, and thus, identified regulated target genes may be different in other cell types. However, the action of many TFs in ES cells is consistent with their normal function in more differentiated cells. For example, MYOD1 activates muscle-specific genes in ES cells which normally happens in myoblasts and myotubes, whereas ASCL1 activates genes specific for neurons [19]. Thus, we expect that many regulated target genes identified in ESCs are functional in differentiated cells. Second, the induction of TF was not complemented by experiments with repression of TFs. Many TFs have high expression in ES cells, and their further induction has either a limited or even inverse effect due to saturation and/or interference. The importance of downregulation of TFs was demonstrated in the large-scale project with mouse ESCs [17], where new relations between TFs and their targets were uncovered in comparison to experiments with TF induction [14, 15]. Third, our approach is focused only on the canonical effect of TFs on target genes via binding to promoters and enhancers. However, there are alternative mechanisms of TF-mediated regulation of gene expression which include cofactor binding, squelching, inactivation, or chromatin modification [44–46]. In addition, the change of gene expression may result from multi-step and/or multi-component regulatory cascades. Analysis of these effects is beyond the limits of this paper. Finally, the experimental system for TF induction is largely artificial (in vitro) and may lack some interactions that exist in vivo, such as cofactor proteins, protein modifications, and epigenetic factors. Thus, the uncovered sets of regulated targets of TFs are not complete and may include some false positives. But despite of these limitations, we believe that our approach is an important step towards better understanding the mechanisms of gene regulation, and our methods should be useful in the future research.

## Conclusions

We developed an atlas of regulated targets of TFs (ART-TF) in human ES cells by combining data on TF binding with a large-scale study of the gene expression change after manipulation of individual TFs. Sets of regulated gene targets were identified for 311 TFs with a controlled rate of false positives. This approach contributes to the understanding of biological functions of TFs and organization of gene regulatory networks. The new atlas should be a valuable resource for understanding the biological functions of TFs and improving ES cell-based regenerative medicine studies.

## Methods

The aim of this study is to identify regulated taerget genes of human TFs in ESCs by combining published information on genome binding of TFs (ChIP-seq data) and gene expression change shortly after induction of each TF. The design is to use gene set enrichment (PAGE) to quantify enrichment of target genes in sets of upregulated and downregulated genes after induction of TFs and evaluate the expected proportion of false positives (EPFP) in sets of regulated targets.

### Assembling data on TF binding sites

ChIP-seq data was extracted mostly from the GEO database [47] (Additional file 1). The majority of ChIP-seq experiments (92.2%) were done with antibody to the TF of interest, other experiments used antibody to tags (FLAG, HA, V5, Biotin) of fused TF genes (GFP, Myc, ER) for immunoprecipitation. We did not find any consistent difference in quality of results if tags or fused genes were used for immunoprecipitation as compared to native antibody, and thus, all data was processed uniformly. One of the TFs, SLBP, functions also as RNA-binding protein; thus we used both ChIP-seq and eCLIP data for analysis. Most ChIP-seq data (> 95%) includes genome coordinates of peaks, as well as scores that characterize the strength of binding, such as MACS [48] output. If scores were not available, we assigned scores equal to one of the following: the number of reads per peak, negative log-transformed p-values, or width of peaks. If peak information was not available, we used other data formats such as wig, bigwig (bw), bedGraph, bed, and bam files. Depending on the input file format, we used a series of Perl programs to identify peaks. Peak coordinates were all converted to human genome hg19 using UCSC LiftOver tool (https://genome.ucsc.edu/cgi-bin/hgLiftOver). Peaks separated by < 500 bp were combined into one. Not more than 25,000 peaks were analyzed in each data set.

ChIP-seq peaks were then associated with transcription start sites (TSSs) of genes using genomic coordinates of RefSeq and ENSEMBL genes (files refGene.gz and ensGene.gz files at http://hgdownload.soe.ucsc.edu/goldenPath/hg19/database/). Alternative TSSs of genes with the same symbol were considered if they were separated by distance > 1 Kb from the main TSS. The shape of peak frequency distribution relative to TSS of all genes was used for quality control of ChIP-seq data. If the cumulative frequency of peaks did not reach a maximum near TSS, we checked if the genome version was correct, which was especially important if the information on the genome version was missing in the GEO database. Each ChIP-seq peak was associated with a maximum of 3 genes whose TSS was within 100 Kb from the peak center. Scores of gene/peak associations were calculated as symbol quality multiplied by the binding score (ChIP-seq) and divided by the distance from the peak to TSS (Kb, capped at 1 Kb). Symbol quality was equal 1 for “weak” symbols (e.g., containing 4 digits in a row, or strings “FAM”, “MIR”, “MRP”, and “orf”) and 3 for normal symbols. Genes with association scores < 20% of the maximum value (i.e., for the best matching gene) were not reported as associated with the given ChIP-seq peak.

Most of analysed ChIP-seq data utilized immunoprecipitation (IP) against TFs used in the experiments with TF-induction [19]. We found and analyzed ChIP-seq data for 302 TFs out of 510 induced TFs. Also, we examined data for additional 13 TFs (35 ChIP-seq data sets) that were not induced but either had a similar binding motif (i.e., belonged to the same gene family) or interacted directly with induced TFs (see “Comparison of methods for delineating regulated target genes of TFs” section).

### Uncovering sets of regulated target genes of TFs

To regulate the expression of target genes, TFs bind to either promoters (proximal sites, < 500 bp from TSS) or enhancers (distal sites, from 0.5 to 100 Kb from TSS). When bound to promoters, TFs regulate transcription by direct interaction with the transcription initiation complex, whereas regulatory effects of enhancer-bound TFs are mediated by enhancer-promoter DNA loop [33]. Because these mechanisms of regulation are different, we generated two sets of target genes for each TF based on their binding to promoters and enhancers, respectively. The score of target genes of a TF was estimated using too methods: (1) as the sum of scores for all binding sites near each gene, and (2) as the maximum score among all binding sites near the gene. Scores of binding sites at promoters did not depend on the distance from TSS because the distance was capped to 1 Kb. Also we used method #3 where binding sites in promoters and enhancers were combined. Eventually we selected method #1 because it yielded a larger number of regulated target genes (see “Comparison of methods for delineating regulated target genes of TFs” section). The number of target genes in each set was limited to 5000 because larger sets of target genes contained more false positives and the final significance of gene set enrichment (“Delineating sets of regulated target genes” section) was lower.

### Delineating sets of regulated target genes

Analysis of regulated target genes is meaningful only if the set of target genes of a TF and the set of regulated genes (e.g., upregulated or downregulated after the induction of the same TF) intersect more than expected by random. Thus, the first step was to evaluate the statistical significance of the association between sets target genes and their regulation. We used the Parametric Analysis of Gene set Enrichment (PAGE) [25], which was selected because of its simplicity and reliability [49]. It determines whether the mean log-expression change, *x*_set_, in genes that belong to a set of target genes, *S*, is significantly greater than expected from the mean and standard deviation of log-expression change in all genes (*x*_all_ and *SD*_all_, respectively). The z-value for testing the null hypothesis is

1 $$z = \frac{{\left( {x_{set} - x_{all} } \right) \cdot \sqrt {n{}_{set}} }}{{SD_{all} }},$$

where *n*_set_ is the number of genes in set *S*. We used ExAtlas [20, 50] to process all sets of target genes and all gene expression data in one step. In ExAtlas, the PAGE method is modified by applying Eq. (1) to the subset of *n* top upregulated genes and another subset of *n* top downregulated genes rather than to all genes. We used the default value: *n* = 1/4 of all genes. To take advantage of scores of association between ChIP-seq peaks and target genes (see “Assembling data on TF binding sites” section), the size of the set of target genes was reduced by increasing gradually the threshold score and repeating the PAGE method for the set of genes with scores higher than the threshold. Then the maximum z-value was used as the final result. This procedure is available in ExAtlas by selecting option “use gene attributes” [20].

If gene set enrichment is statistically significant (*p* ≤ 0.05), then ExAtlas estimates the expected proportion of false positives (EPFP) for each target gene that changed expression by more than a threshold value (we used 1.5-fold threshold). EPFP equals the proportion of targets among “control” genes that are presumably not affected by TF manipulation (which changed by < 1.2 fold) divided by the proportion of targets among genes that responded to TF induction stronger than the given gene [11]. EPFP values are then adjusted making them increase monotonically with the decreasing expression change of genes. Then genes with EPFP below the accepted level (in our case, EPFP = 0.3), comprise the set of regulated target genes. Sets of regulated targets obtained with different ChIP-seq experiments were then combined, and the lowest EPFP value was assigned to each target gene. Regulated target genes supported by only a single ChIP-seq experiment were excluded from the final list, except for 75 TFs where only a single ChIP-seq data set was successful in generating some regulated target genes. In Fig. 3A–F we used ChIP-seq data sets for 6 TFs: CEBPB-20, CEBPB_24, JUN_05, JUN_13, KLF4_02, KLF4_06, MYC_04, MYC_17, REST_07, REST_10, TEAD4_04, and TEAD4_15.

## Supplementary Information

**Additional file 1:** ChIP-seq data used for identifying regulated targets of human transcription factors.

**Additional file 2:** Regulated targets of transcription factors identified based on each ChIP-seq data set. The second worksheet includes counts of ChIP-seq data sets that yielded some regulated target genes for each TF.

**Additional file 3:** Surrogate ChIP-seq data for finding indirect regulated targets of transcription factors.

**Additional file 4:** Number of regulated target genes of individual transcription factors combined from multiple ChIP-seq data sets.

**Additional file 5:** Table of all regulated targets of TFs.

**Additional file 6:** Comparison of lists of regulated target genes generated here (ART-TF) with existing databases.

**Additional file 7:** Similarity of sets of regulated target genes of most strongly overlapping 150 TFs in ESCs; z-values show the significance of overlap between gene sets (hypergeometric test).

**Additional file 8:** Similarity of sets of regulated target genes of TFs in ESCs; z-values show the significance of overlap between gene sets (hypergeometric test).

**Additional file 9:** User’s guide for accessing ART-TF data in ExAtlas.

## Data Availability

Input ChIP-seq data are available in the GEO repository, https://www.ncbi.nlm.nih.gov/geo/; data series and sample ID are specified in Additional file 1. Input data on gene expression profiles of ES cells after induction of TFs are available in the ExAtlas repository, http://alexei.nfshost.com/exatlas/, data set: public-CREST_Human_TF_induction_ESC_20181203. Most results generated during the current study are available either in Additional Files or in the ExAtlas repository (see link above). A user’s guide for accessing ART-TF data in ExAtlas is in Additional file 9. ExAtlas source code and other programs for data analysis are available at Github (https://github.com/AlexeiSharovBaltimore/ExAtlas and https://github.com/AlexeiSharovBaltimore/ART-TF). The README file in the ART-TF Github project explains the overall dataflow.

## Footnotes

1 For each ChIP-seq data set, all regulated targets were combined: upregulated, downregulated, bound by TFs in enhancers and promoters.
