# Supplementary material for: Atlas of regulated target genes of transcription factors (ART-TF) in human ES cells
Source: BMC Bioinformatics. 2022 Sep 16;23:377. doi: 10.1186/s12859-022-04924-3 (PMC9479252; doi:10.1186/s12859-022-04924-3)
Supplement: Supplementary file 9 — Additional file 9: User’s guide for accessing ART-TF data in ExAtlas. [file 12859_2022_4924_MOESM9_ESM.doc]

**Additional file 9. User’s guide for accessing ART-TF data in ExAtlas**

ExAtlas is a software for meta-analysis of gene expression data (Sharov et al. 2015; Sharov and Schlessinger 2018). Besides standard statistical analysis of gene expression (similar to NIA Array Analysis) it supports several methods for meta-analysis and generates results for all combinations of data in multi-component data sets (e.g., all gene expression profiles vs. all GO annotations). It includes standard meta-analysis: fixed and random effects (DerSimonian-Laird), z-score and Fisher's methods; global correlation analysis between different gene expression data sets; gene set enrichment among upregulated and downregulated genes; gene set overlap; gene association (e.g., finding sets of coregulated genes in two similar cell types of tissues or regulated targets of transcription factors). Expression profile data can be uploaded manually or extracted from the Gene Expression Omnibus (GEO) database. In particular, users can combine samples from multiple data sets (and possibly different platforms) in GEO database, assess the quality of data, and perform statistical analysis. Preloaded public data includes most popular public data sets (e.g., GNF, BrainScope, Gene Ontology, KEGG, GAD phenotypes).

ExAtlas is available at http://alexei.nfshost.com/exatlas/ and the code can be accessed at https://github.com/AlexeiSharovBaltimore/ExAtlas. Users can login as guests by clicking the button “Start using ExAtlas”. To access ART-TF data, users need to select organism species by clicking the pull-down menu “Select organism” shown by blue arrow at the top of the screen. Please, select “Human (Homo sapiens)”. In the section “Select Data Files” on the second line locate a pull-down menu that by default shows data “public-ART-TF_regulated_targets_hg19_add_all”. The name of the data set indicates that human genome hg19 is used; target genes of TFs were idendified by “adding” scores of associated binding sites; and “all” means that both direct and indirect (i.e., surrogate) ChIP-seq data were used.

Open the pull-down menu to see 5 files with ART-TF project results:

1. public-ART-TF_regulated_targets_hg19_add_all = Combined direct and indirect regulated targets with binding in promoters and enhancers of 311 TFs.

2. public-ART-TF_regulated_targets_hg19_add_direct = Direct regulated targets with binding of TFs in promoters and enhancers.

3. public-ART-TF_regulated_targets_hg19_add_enhancers = Combined direct and indirect regulated targets with binding of TFs in enhancers.

4. public-ART-TF_regulated_targets_hg19_add_promoters = Combined direct and indirect regulated targets with binding of TFs in promoters.

5. public-ART-TF_targets_hg19_enhancers_add = Targets of TFs with binding in enhancers.

6. public-ART-TF_targets_hg19_promoters_add = Targets of TFs with binding in promoters.

Select the first file “public-ART-TF_regulated_targets_hg19_add_all” and click a button “Open” in the same line at the left. The next screen shows a histogram that indicates the numbers of regulated target genes for transcription factors (TFs). Orange and blue bars indicate sets of target genes that are upregulated and downregulated, respectively, after the induction of a corresponding TF in ES cells. To display sets of regulated target genes, use section (1) where users can search for TFs or select them from a pull-down menu. For example, select “ASCL1_up”, meaning upregulated target genes after induction of ASCL1 and then click the button “Display genes”. This will bring a table of regulated target genes with attributes such as EPFP, nData = number of supporting ChIP-seq data, logratio (log10) of gene expression change after induction of a TF, predicted position of the binding site (with maximum score) and gene name. The link named “Table of genes” above the table can be used to download the table as tab-delimited text to be analyzed in Excel or other software. Below this link there is a tool for quick analysis of gene set overlap. By default, the GO annotations gene set is selected; thus, by clicking the button “Overlap analysis” users can immediately find which GO annotations are overrepresented in the table of genes. When the results appear, click the button “Get profile” (in section 2) to display enrichment statistics (histogram of z-values) and a table of enriched GO-terms. The top is “GO:0045746, negative regulation of Notch signaling pathway”, which indicates that ASCL1 contributes to activating Notch signaling.

Now let’s return back to the screen where the data set “public-ART-TF_regulated_targets_hg19_add_all” was open. To search for all TFs that activated gene GATA6, type in this gene symbol in the search box, at the left pull-down menu change “Find genesets” to “Gene symbols”, and then click “Search”. Results indicate that 21 gene sets include GATA6.

To generate a matrix of similarity between gene sets use section “2. Analyze overlap with another geneset file”. In this exercise, we will compare the data set “public-ART-TF_regulated_targets_hg19_add_all” with itself. Thus, in the line “Select file” use the pull-down menu to select the same data set, then click “Overlap analysis” button. After the task is finished click the button “Check your task”, and in the following screen click the button “Plot output table”. The result is a color-coded matrix which shows the enrichment of gene set overlap for target genes of all TFs, upregulated and downregulated. This matrix can be downloaded as tab-delimited text and used for further analysis (use link “Matrix file”).

**References**

Sharov AA, Schlessinger D, Ko MS: ExAtlas: An interactive online tool for meta-analysis of gene expression data. J Bioinform Comput Biol 2015, 13(6):1550019.

Sharov AA, Schlessinger D: ExAtlas: On-line tool to integrate gene expression and gene set enrichment analyses. In: Molecular-Genetic and Statistical Techniques for Behavioral and Neural Research. Edited by Gerlai RT. San-Diego: Academic Press; 2018: 73-193.
